# What Rankings, Ratings, and Utilities Do Breast Cancer Patients Place on Tissue- and Implant-based Breast Reconstruction?

**DOI:** 10.1097/GOX.0000000000006749

**Published:** 2025-05-01

**Authors:** Haoqi Wang, Nsikak M. Jackson, Danmeng Huang, Rachel E. Alexander, Mary Catherine Bordes, Jun Liu, Tzuan A. Chen, Fatima Merchant, Summer E. Hanson, Mark Schaverien, Mia K. Markey, Gregory P. Reece, Scott B. Cantor, Aubri S. Hoffman

**Affiliations:** From the * Department of Biomedical Engineering, The University of Texas at Austin, Austin, Texas; † Department of Plastic Surgery, The University of Texas MD Anderson Cancer Center, Houston, Texas; ‡ Department of Health Services Research, The University of Texas MD Anderson Cancer Center, Houston, Texas; § Department of Engineering Technology, University of Houston, Houston, Texas; ¶ HEALTH Research Institute, University of Houston, Houston, Texas; ∥ Department of Psychological, Health, and Learning Sciences, University of Houston, Houston, Texas; ** Department of Surgery, The University of Chicago, Chicago, Illinois; †† Department of Imaging Physics, The University of Texas MD Anderson Cancer Center, Houston, Texas.

## Abstract

**Background:**

Utility analysis is well-established for comparing treatment options but challenging to assess with patients in clinical care. Preference assessment may be more feasible, but it is not yet known whether it correlates with utilities. The aims of this study were to (1) assess women’s rankings, ratings, and utilities for tissue-based, implant-based, and no reconstruction after total mastectomy; and (2) explore assessment methods and correlations with clinical and psychosocial factors.

**Methods:**

Forty patients considering or undergoing breast reconstruction completed 3 assessments—card ranking, visual analog scale, and standard gamble—and psychosocial questionnaires. Each woman rated 9 health states with case-matched images: 4 excellent, good, fair, and poor outcomes for tissue-based reconstruction; 4 for implant-based reconstruction; and 1 image for no reconstruction. Nonparametric tests compared assessment methods. Descriptive statistics summarized rankings, ratings, and utilities. Multivariable regression models assessed correlations with clinical/psychosocial factors.

**Results:**

Median standard gamble utilities by category were 0.97 (excellent), 0.95 (good), 0.94 (fair), and 0.92 (poor) for tissue-based reconstruction; 0.99 (excellent), 0.96 (good), 0.94 (fair), and 0.94 (poor) for implant-based reconstruction; and 0.86 for no reconstruction. The standard gamble required 20–40 minutes, and some patients found it difficult. The visual analog scale required 5 minutes and correlated with the standard gamble. Psychosocial characteristics correlated with the scores; however, a more diverse sample is needed.

**Conclusions:**

All assessments showed that women highly value breast reconstruction after mastectomy. For time-limited clinical care, the visual analog scale is brief, understandable, and clearly illustrates preferences to support shared decision-making.

**Takeaways**

**Question:** What preferences and utilities do women undergoing mastectomy place on breast reconstruction (overall, and for tissue- and implant-based reconstruction), how do psychosocial factors impact ratings, and which assessment methods are feasible for clinical care?

**Findings:** Upon completing card ranking, visual analog scale, and standard gamble activities, participants reported high utilities and ratings for all outcomes, moderated by psychosocial factors. Nearly half of the participants reported unexpected preferences, emphasizing the need for shared decision-making.

**Meaning:** Clinicians can consider using a visual analog scale to assess preferences (it correlates well with utilities, is quick, and is more intuitive) and consider the impact of key psychosocial factors.

## INTRODUCTION

Health utilities are a key measure of patients’ strength of preference for various health states, summarizing both positive and negative aspects into 1 score between 0 (death) and 1 (perfect health). They are used at the population level to inform policy and reimbursement, but can be time-intensive and challenging to implement or interpret in clinical care with individual patients. As a result, previous studies of the utility of breast reconstruction^1–5^ relied on assessments provided by surgeons or healthy individuals,^1–5^ leaving a gap in the evidence. Alternatively, preference assessment methods, such as inviting patients to rank or rate outcomes, may be more feasible; however, it is unknown whether rankings and ratings correlate with utilities in the context of breast reconstruction. Further, prior studies^1–5^ assessed the utility of reconstruction versus no reconstruction, but not tissue- versus implant-based reconstruction.

Our long-term goals are to provide scientists and policymakers with patient-generated normative utility information and to provide clinicians and patients with a feasible, easy-to-use patient decision aid.^6–9^ As a formative step, this study sought to address the gap in evidence by collecting and reporting patient-reported utilities for tissue-based, implant-based, and no breast reconstruction. Secondary goals were to measure correlations among patient-reported utilities and participants’ rankings, ratings, psychosocial factors, and characteristics, while exploring the feasibility and timing of assessment within the clinical care flow.

## METHODS

Guided by our multidisciplinary patient and stakeholder advisory panel, we engaged patients who were considering or undergoing breast reconstruction in completing the 3 gold-standard preference and utility assessments—card ranking, visual analog scale, and standard gamble—and questionnaires assessing their characteristics, quality of life, and appearance investment. The institutional review board at The University of Texas MD Anderson Cancer Center approved this study.

### Subjects and Setting

Participants were patients whose legal sex was female (hereafter referred to as women per their preference); aged 21 years or older; able to read and speak English; and considering or undergoing breast reconstruction after total mastectomy, as identified in the medical record. All participants provided informed consent. To address the question of whether the measures correlated and were feasible in clinical consultation, a sample size of 40 women was selected, congruent with similar studies in the extant literature.^10^

### Breast Reconstruction Decisions

During a consultation visit, participants were given information about 3 reconstruction options (tissue-based, implant-based, or none) that emphasized that there are no differences in recurrence rates or ability to detect a recurrence among the options, and that there are meaningful differences in the treatment process, lived experience, and aesthetic outcomes.^11–14^ Participants were also informed about potential revision surgery, nipple reconstruction, and tattoos.

### Recruitment and Baseline Questionnaires

A trained interviewer (N.M.J.) recruited participants by telephone and conducted 90-minute interviews in a private room. To assess the feasibility and effect of assessment timing, we alternated recruiting during the premastectomy decision-making process and after initial breast surgery.

Participants responded to a questionnaire assessing sociodemographic and clinical characteristics, BREAST-Questionnaire (BREAST-Q, v. 2.0^15^), and Appearance Schemas Inventory—Revised (ASI-R^16,17^). BREAST-Q is a patient-reported outcome measure assessing quality of life and satisfaction,^15^ scored on a 0–100 scale, with higher scores indicating greater well-being and satisfaction (Cronbach alpha 0.81–0.96). ASI-R is an empirically validated measure of psychological investment in one’s physical appearance,^17^ with 3 subscales: appearance self-evaluation, appearance power/control, and appearance standards/behaviors. Items are rated on a scale of 1–5, with higher scores indicating more investment (Cronbach alpha 0.82–0.91).

### Ranking, Rating, and Utility Assessment

Participants completed 3 activities assessing their ranking, rating, and utility of breast reconstruction outcomes. The interviewer provided participants with a set of 9 cards, case-matched to their body mass index (BMI) category (normal, overweight, and obese) and reconstruction plan (unilateral or bilateral). (**See figure, Supplemental Digital Content 1**, which displays the example image and health state cards [fronts and backs] for women with unilateral breast cancer who are overweight and considering bilateral breast reconstruction, http://links.lww.com/PRSGO/E7.) Four cards illustrated excellent, good, fair, and poor outcomes of implant-based reconstruction. Four cards illustrated excellent, good, fair, and poor outcomes of tissue-based reconstruction. One card illustrated no reconstruction. Images were selected by a board-certified reconstructive surgeon (G.P.R.) from a dataset of more than 500 patients (The University of Texas MD Anderson Cancer Center protocol number 2010-0321, supported by R01 CA143190).

Each card provided a health state description of the lived experience. These descriptions were systematically codeveloped and pilot-tested with survivors, expressing in plain language the factors patients deemed most important when making decisions about breast reconstruction, such as confidence in their appearance, effects on social engagement, satisfaction with sex life, and overall satisfaction with the outcome.

#### Card Ranking

The interviewer invited each participant to review the health state descriptions and images. The interviewer then invited the participant to rank the potential outcomes from most preferred (1) to least preferred (9).

#### Visual Analog Scale Rating, With and Without Death as an Option

Next, participants rated the potential outcomes using a visual analog scale or “feeling thermometer” from 0 (least preferred) to 100 (most preferred). To assess the correlation between visual analog scale ratings and standard gamble utilities (which involve the chance of death), the interviewer added a card that said “death” as a potential outcome and asked them to repeat the card ranking and visual analog scale rating. The interviewer explained that they could place the death card at 0 if they felt death was the least preferred outcome, or they could place another card at 0 and place death on the scale at the point they felt indicated how much preferable death was to the other health states.^18^

#### Standard Gamble

The standard gamble^19^ is a classical method of measuring cardinal preferences for health states related to the theories by Von Neumann and Morgenstern.^20^ It asks: “What percent risk of immediate death (or least preferred outcome) would a participant be willing to accept for perfect health (or most preferred outcome)?” Participants are offered a theoretical choice between a 100% certainty of an intermediate outcome and a gamble between their most and least preferred outcomes (or perfect health and death). For example, the interviewer may start by offering a 100% chance of a good reconstruction versus a gamble between a 95% chance of an excellent reconstruction and a 5% chance of a poor reconstruction. If the participant chooses the gamble, the interview repeats the question, offering 100% good versus a gamble of 90% excellent and 10% poor outcomes.

The interviewer demonstrated the activity using blindness as an example and then led each participant through the standard gamble. The gamble started with a probability, *P*, of 100% for their best outcome and 1 − *P* for their worst outcome. The interviewer iteratively lowered the probability of the best outcome until the participant selected the certainty of the intermediate outcome. The process was repeated for all 7 intermediate outcomes. Because participants were facing a potentially terminal diagnosis, the interviewer repeated the process using the chained gamble method,^21^ in which the participant chooses between the certainty of each intermediate outcome and a gamble between their most preferred outcome and death.^22^

### Feasibility

To assess the feasibility of conducting these assessments as part of the clinical care flow, the interviewer noted any patient-reported concerns, difficulties completing the assessments, and time needed.

### Statistical Analysis

Descriptive statistics summarized the rankings, ratings, and utilities. The Spearman rho and Wilcoxon signed ranks explored correlations between assessment methods and reconstruction options. Linear regression models explored associations among visual analog scales and body investment, appearance investment, and other characteristics. All analyses were performed in R (version 4.2.2)^23^ by a trained analyst and reviewed by a second statistician (H.W. and T.A.C).

For this study, a utility represents the participant’s evaluation of quality of life associated with a particular outcome. To address the primary objective, medians and interquartile ranges summarized nonnormal distributions. All utility values were calculated from 0.0 (least preferred) to 1.0 (most preferred). In the first step of the chained gamble,^19^ the utility of a given outcome, *u* (outcome), was calculated as follows:


$$u\left(\text{outcome}\right)=\text{[}P_{1\text{i}}\times u\left(\text{most}\text{preferred}\text{outcome}\right)]+\left(1-P_{1i}\right)\times \text{u}\left(\text{least}\text{preferred}\text{outcome}\right).$$

In the second step, the utility of the least preferred outcome assessed with a gamble between the most preferred outcome, and death was calculated as follows:


$$\begin{array}{l} u\left(least\;\;\;preferred\;\;\;outcome\right)=P_{2}. \end{array}$$

Combining the formulas, the utility of any given outcome was estimated as follows:


$$\begin{array}{l} u\left(outcome_{i}\right)=P_{1i}+\left(\left(1-\;P_{1i}\right)\times P_{2}\right). \end{array}$$

Spearman rank correlations assessed correlations between utilities and rankings. Wilcoxon signed-rank tests compared ratings and utilities overall, between options, and between reconstruction quality levels (excellent, good, fair, poor), adjusted for within-participant variations. Kruskal–Wallis tests compared ratings between BMI categories.

Univariate and multivariable regression models assessed correlations with participants’ psychosocial factors and characteristics. Covariates with a *P* value less than 0.2 in the univariate analysis were retained. Backward model selection based on Akaike information criterion^24^ was used.

To assess feasibility, we tabulated the number of participants who were willing and able to successfully complete the assessment, the time to completion, and any perceived or expressed difficulties. All tests were 2-sided. *P* values less than 0.05 were considered statistically significant.

## RESULTS

### Participant Characteristics

The majority of the 40 participants (Table 1) reported being 27–68 years old, non-Hispanic (78%), White (92%), and married (78%). Six participants underwent prophylactic bilateral mastectomy. Among 24 participants (60%) considering bilateral reconstruction, 15 (62.5%) underwent mastectomy and reconstruction of an unaffected breast for symmetry.

**Table 1.** Participant Characteristics (n = 40)

Sociodemographic Characteristics	n, %	
Age, y, mean ± SD (min, max)	51.65 ± 11.66 (27, 68)	
Race		
White	37, 92	
Asian	1, 3	
Black	1, 3	
Other	1, 3	
Hispanic	9, 23	
Married	31, 78	
Highest level of education		
High school or some college	13, 33	
College graduate or higher	27, 68	
Clinical characteristics		
Indications for mastectomy		
Cancer in 1 breast	30, 75	
Cancer in both breasts	5, 13	
Prophylactic for 1 breast	9, 23	
Prophylactic for both breasts	6, 15	
Reconstruction laterality		
Unilateral	16, 40	
Bilateral	24, 60	
Weight status		
Normal weight (BMI: 18.5–24.9 kg/m^2^)	12, 30	
Overweight (BMI: 25.0–29.9 kg/m^2^)	16, 40	
Obese (BMI: 30.0 kg/m^2^ and above)	12, 30	
Psychological characteristics	Mean (SD)	Min, Max
ASI-R	3.28 (0.59)	1.95, 4.45
Behavior	3.76 (0.63)	2.14, 5.00
Self-evaluation	2.97 (0.77)	1.25, 4.50
Control	3.09 (0.65)	1.80, 4.40
BREAST-Q		
Breast	54.11 (9.90)	35.75, 74.00
Outcome	57.99 (13.68)	32.00, 80.00

### Assessment Methods and Timing

Card ranking took an average of 4 minutes (minimum 1 and maximum 6), without any concerns. Most participants briefly read the health state descriptions, then focused on the images. The results could be easily interpreted, but could not illustrate participants’ strength of preference for each option. Interestingly, although there was some concern from the clinical team about including images with poor outcomes, participants expressed appreciation for seeing realistic examples from a trusted source and reported that the images were less scary than what they viewed online.

Visual analog scale rating took an average of 3 minutes (minimum 1 and maximum 5), and only 1 woman had difficulty. Without death, all participants placed their most preferred card at 100 and least preferred card at 0 without prompting, and dispersed the rest, making it easy to identify the strength of preference for each option. When the death card was introduced, most participants clustered their ratings at or near 100, making it difficult to visually distinguish the options.

The standard gamble took an average of 12 minutes (minimum 9 and maximum 17), and 9 participants had questions or concerns about the process and length of time. One record was dropped, as the participant had to leave before completing the assessments.

### Card Ranking

Overall, participants’ rankings were consistent with surgeons’ ratings of excellent, good, fair, and poor outcomes (Table 2). However, at the individual level, participants’ preferences varied in terms of their highest and lowest ranking, with 18 participants (45%) providing an unexpected ranking (ie, a fair outcome ranked in their top 3 or an excellent outcome in their bottom 3). Ten participants (25%) verbalized a preference for 1 or both excellent options and equipoise among the remaining options except death.

**Table 2.** Patient-reported Rankings, Ratings, and Utilities for Any, Implant-based, Tissue-based, and No Breast Reconstruction: Medians and Interquartile Ranges

	Card Ranking, 1 (Most Preferred) to 9 (Least Preferred)	Visual Analog Scale, 100 (Most Preferred) to 0 (Least Preferred)		Standard Gamble, 1.0 (Most Preferred) to 0 (Least Preferred)	*P * *
Potential Outcomes, Median (IQR)	Without Death as an Option (n = 40)	Without Death as an Option (n = 40)	With Death as an Option (n = 39)	With Death as an Option (n = 39)	Visual Analog Scale With Death Versus Standard Gamble
Any reconstruction	4.5 (0.0)	56.6 (10.9)	96.3 (26.9)	0.94 (0.06)	0.006
Excellent	2.0 (1.1)	87.5 (18.1)	100 (5.0)	0.97 (0.03)	0.010
Good	3.8 (1.5)	70.0 (21.3)	100 (22.5)	0.96 (0.07)	<0.001
Fair	5.5 (1.0)	40.0 (31.3)	92.5 (32.5)	0.94 (0.09)	0.004
Poor	7.0 (1.1)	27.5 (23.4)	95.0 (40.0)	0.93 (0.12)	0.019
Implant-based reconstruction	4.3 (0.8)	59.4 (14.6)	95.0 (20.6)	0.95 (0.05)	0.008
Excellent	2.0 (2.0)	95.0 (30.0)	100 (0.0)	0.99 (0.03)	0.072
Good	3.0 (2.3)	75.0 (35.0)	100 (15.0)	0.96 (0.06)	<0.001
Fair	5.0 (3.0)	50.0 (42.5)	100 (30.0)	0.94 (0.06)	0.014
Poor	7.0 (1.0)	27.5 (35.5)	100 (40.0)	0.94 (0.08)	0.018
Tissue-based reconstruction	4.8 (1.0)	52.5 (19.6)	94.5 (28.9)	0.94 (0.08)	0.003
Excellent	2.0 (2.0)	90.0 (20.0)	100 (0.5)	0.97 (0.06)	<0.001
Good	4.0 (2.0)	65.0 (30.5)	100 (30.0)	0.95 (0.07)	<0.001
Fair	6.0 (2.0)	34.0 (30.8)	100 (45.0)	0.94 (0.13)	<0.001
Poor	8.0 (2.0)	20.0 (24.0)	90.0 (40.0)	0.92 (0.17)	0.025
No reconstruction	9.0 (0.0)	0 (0.0)	0 (0.0)	0.86 (0.15)	0.563
Reconstruction versus no reconstruction*, P*†	<0.001	<0.001	<0.001	<0.001	
Implant versus tissue*, P*†	<0.003	0.005	0.122	<0.001	

* *P* values assessed using Spearman correlation test; *P* < 0.05 indicates significantly correlated median scores.

† *P* values assessed using paired Wilcoxon signed rank test; *P* < 0.05 indicates significantly different median scores.

IQR, interquartile range.

### Visual Analog Scale Ratings

**Fig. 1.**
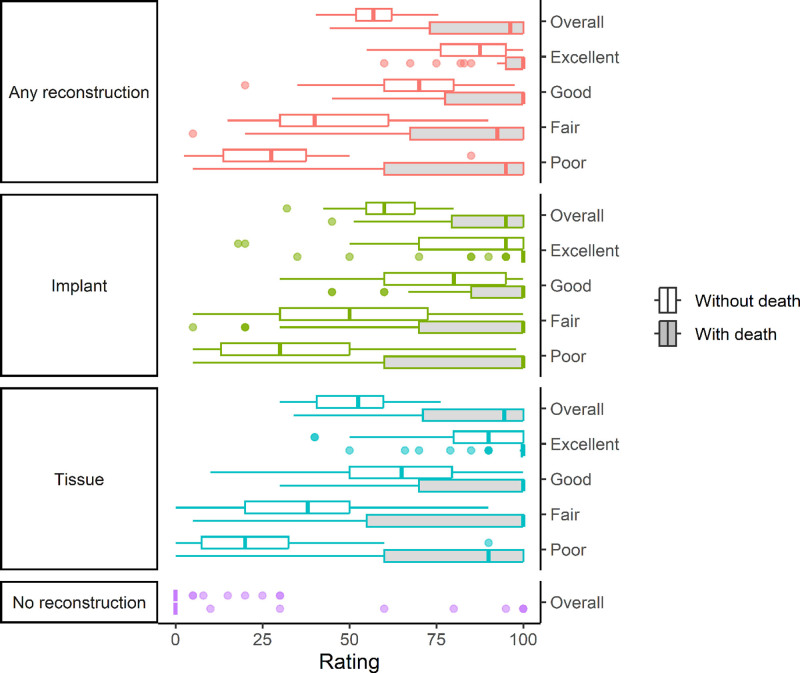
Median and interquartile ranges of participants’ visual analog scale ratings (with and without death as an option) for any, implant-based, tissue-based, and no reconstruction.

Without death included as an option, the median and mean visual analog ratings were similar (57 versus 56, respectively); yet again, ratings varied widely at the individual level, with 15 participants clustering their ratings in 2 groups—near 100 and near 0 (Table 2 and Fig. 1). Notably, 30 out of 39 participants (77%) rated their top 2 options above 90, indicating that many patients may have strong preferences for 2 (or more) options.

When death was included, the majority of participants shifted their ratings toward 100, as expected, with 17 (44%) of the participants rating all 9 options at or near 100. However, 23 participants (59%) provided at least 1 unexpected rating, including 1 rating of no reconstruction as less preferable than death. Notably, although the median rating for no reconstruction was 0, 9 participants (23%) provided a nonzero rating for no reconstruction.

### Standard Gamble Utilities

**Fig. 2.**
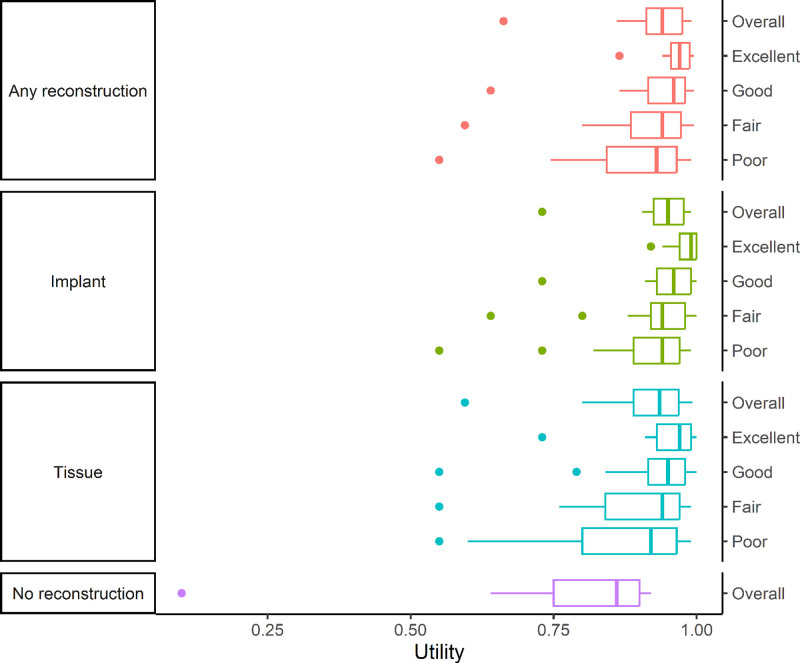
Median and interquartile ranges of participants’ standard gamble utilities (with death as an option) for any, implant-based, tissue-based, and no reconstruction.

Median utilities were high (Table 2, with variance illustrated in Fig. 2) with all reconstructions exceeding 0.90 and no reconstruction at 0.86. Yet even when a chance of death was part of the gamble, participants reported utilities as low as 0.73 for excellent reconstruction, 0.55 for poor reconstruction, and 0.10 for no reconstruction. Overall, patient-reported utilities aligned with surgeon ratings of excellent, good, fair, and poor; however, once again, a notable amount of variation was observed at the individual level, with 49% of participants reporting an unexpected option.

### Comparing Reconstruction Options

Overall, median utilities for implant- and tissue-based reconstruction appeared similar (0.95 versus 0.94) (Table 2), yet they were significantly different (*P* < 0.001) owing to the underlying skewed and clustered distributions in individual preferences. Twenty-seven (69%) of the 39 participants ranked an implant-based reconstruction the highest, and 20 participants (53%) reported the highest utility of 1.0 for implant-based reconstruction. Thirty-two participants (81%) chose no reconstruction as their lowest rank, whereas 8 participants (21%) chose a fair or poor tissue-based reconstruction.

### Exploring Associations With Psychosocial, Sociodemographic, and Clinical Characteristics

#### Visual Analog Scale Ratings, Without Death as an Option

Univariate analyses of visual analog scale ratings without death indicated correlations with BREAST-Q, ASI-R, BMI, reconstruction laterality (unilateral or bilateral), and assessment timing. Age, sex, race, ethnicity, marital status, and education were not significantly correlated. In multivariable models, BREAST-Q was significantly correlated (Table 3); however, the effects were small. ASI-R had larger effects on reconstruction ratings, with every 1-point increase in ASI-R self-evaluation score decreasing reconstruction ratings by 6.96 out of 100 points (*P* ≤ 0.001).

**Table 3.** Multivariable Regression Models of Psychosocial Characteristics

	Any Reconstruction			Implant-based Reconstruction			Tissue-based Reconstruction			No Reconstruction		
	*B*	CI	*P*	*B*	CI	*P*	*B*	CI	*P*	*B*	CI	*P*
Visual analog scale,* without death												
BREAST-Q, breast	0.29	0.08–0.51	0.009	0.46	0.14–0.79	0.006						
BREAST-Q, outcome										−0.25	−0.44 to −0.07	0.008
ASI-R behavior	4.20	0.11–8.30	0.044									
ASI-R self-evaluation	−6.96	−10.15 to −3.78	<0.001				−6.04	−10.52 to −1.56	0.010			
Cancer in 1 breast												
Overweight												
Obese				−16.15	−23.76 to −8.53	<0.001	10.59	2.17–19.02	0.015			
Assessed after mastectomy												
Visual analog scale,* with death												
BREAST-Q, breast												
BREAST-Q, outcome										−0.90	−1.64 to −0.16	0.018
ASI-R behavior												
ASI-R self-evaluation												
Cancer in 1 breast	16.55	2.83–30.27	0.020	14.06	1.04–27.08	0.035	18.53	4.69–32.38	0.010			
Overweight												
Obese				−13.79	−27.59 to 0.01	0.050						
Assessed after mastectomy												
Standard gamble,† with death												
BREAST-Q, breast												
BREAST-Q, outcome												
ASI-R behavior												
ASI-R self-evaluation												
Cancer in 1 breast	0.11	0.03–0.18	0.007	0.08	0.02–0.14	0.009	0.13	0.04–0.22	0.007	0.24	0.04–0.45	0.018
Overweight												
Obese												
Assessed after mastectomy												

* Visual analog scale ratings, scored 0 (least preferred) to 100 (most preferred).

† Standard gamble utilities, scored 0 (least preferred) to 1.0 (most preferred).

B, regression coefficient, represents the estimated change in the dependent variable for a 1-unit increase in the independent variable, while holding all other independent variables constant; CI, confidence interval.

**Fig. 3.**
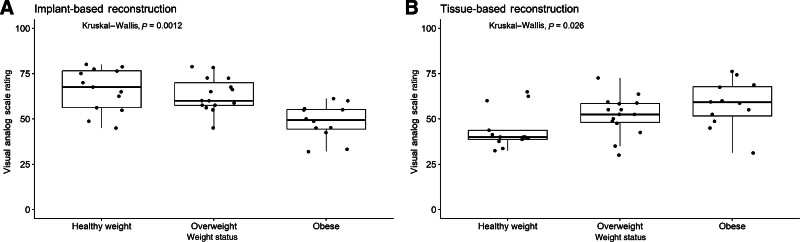
Median and interquartile ranges of participants’ visual analog scale ratings of implant-based (A) and tissue-based reconstruction outcomes by BMI (B).

As weight status increased (Fig. 3), median ratings of implant-based reconstruction decreased, whereas ratings of tissue-based reconstruction increased. Relative to participants with healthy weight, obesity significantly decreased median ratings for implant-based reconstruction by 16.15 points and increased ratings for tissue-based reconstruction by 10.59 points. Notably, participants with a healthy weight who participated during their decision-making process reported a median rating of 56.63 out of 100 for tissue-based reconstruction, whereas participants who participated after their initial breast surgery reported a median rating of 48.13 (*P* = 0.009).

#### Visual Analog Scale Ratings, With Death as an Option

In univariate analyses, visual analog scale ratings with death as an option correlated with mastectomy indication (cancer or prophylactic), reconstruction laterality, BMI, BREAST-Q, ASI-R, and assessment timing. In multivariable analyses (Table 3), mastectomy indication and laterality were significantly correlated, such that participants with cancer in 1 breast rated implant-based reconstruction 14.06 points higher (*P* = 0.035) and tissue-based reconstruction 18.53 points higher (*P* = 0.010) than the participants with bilateral or prophylactic indications.

#### Standard Gamble Utilities

For utilities, BREAST-Q, ASI-R, and mastectomy indication were significant in univariate models. However, parsimonious models retained only mastectomy indication (Table 3), such that having breast cancer in 1 breast was significantly associated with small but higher utilities for all reconstruction options, including no reconstruction. No differences were observed when utilities were assessed during participants’ initial decision-making process versus after initial reconstruction.

## DISCUSSION

### Main Results

Patient-reported median utilities were 0.94 and 0.86 for reconstruction and no reconstruction, and 0.95 and 0.94 for implant-based and tissue-based reconstruction, respectively. The visual analog scale was more efficient, visually illustrated the distributions of participants’ preferences, and correlated with the standard gamble utilities.

Overall, patient-reported utilities and ratings aligned with surgeon’s ratings of excellent, good, fair, and poor outcomes on average; however, individual participants’ utilities and ratings varied widely, with over half of the participants providing an unexpected rating (a low rating for an excellent outcome or high rating for a poor outcome). Several participants placed very high importance on breast reconstruction, even when explicitly compared with a risk of death. Participants with unilateral breast cancer and higher quality of life scores reported higher ratings, whereas participants who reported more time spent on appearance-fixing behaviors reported lower ratings. Higher BMI increased ratings of tissue-based reconstruction and decreased ratings of implant-based reconstruction.

### Implications for Clinical Counseling

Women highly value breast reconstruction after mastectomy. In time-limited consultations, clinicians can use the brief, intuitive visual analog scale to assess preferences and help patients compare options, with more in-depth shared decision-making for patients with higher BMI or those who self-report higher attendance to their appearance to address unrealistic expectations (eg, assumptions that tissue-based reconstruction includes a “free tummy tuck”). Further, combining shared decision-making with approaches, such as motivational interviewing, may help address misinformation, foster realistic expectations, and improve quality of life.

### Implications for Research and Policy

The results of this study provide evidence of the high utility placed on breast reconstruction and the significant prevalence of unexpected ratings, which did not differ by age, race, ethnicity, or level of education. They align with studies of patients’ clinical and psychosocial characteristics and satisfaction.^6,11–15,25^ Researchers may wish to explore which aspects of the images influenced participants’ utility ratings, particularly for the outliers.

Although US federal laws mandate coverage for breast reconstruction, multiple studies have reported unwarranted variation in the use of autologous and implant-based reconstruction.^26–28^ Results from this study emphasize the preference-sensitive nature of breast reconstruction decisions, and the impact of these decisions on mental health, quality of life, and recovery are well documented. Legislation is needed to ensure high-quality shared decision-making.

### Limitations

Studies are needed to explore patient-reported utilities and rankings for galleries of photographs matched to cancer stage and treatment plan; for other outcomes, such as failed reconstruction; in other groups, such as patients undergoing bilateral or partial mastectomy; and across multiple countries, races, and languages. A larger sample size is needed to assess interactions between complex psychosocial and decision-making factors, and longitudinal effects.^29–31^ Further studies are needed that include a spectrum (poor, fair, good, and excellent) for no reconstruction. Additionally, for the purpose of the study, participants repeated the assessment without death and then with death, which may have introduced an ordering bias. Conducting a single assessment in a clinical consultation or patient decision aid may produce different results.

## CONCLUSIONS

Women report high value for breast reconstruction as a key part of their breast cancer care—even compared with death. These patient-generated utilities can be used to inform cost–utility analyses to guide policy and reimbursement. Notably, decisions between implant-based and tissue-based reconstruction remain highly preference-sensitive. The shorter, more intuitive visual analog scales correlated with utilities and can be used to guide shared decision-making to ensure each woman receives the best option for her.

## DISCLOSURES

The authors have no financial interest to declare in relation to the content of this article. This study was supported in part by funding from the National Cancer Institute (NIH R01 CA203984, multiple principal investigators Markey, Merchant, and Reece) and the National Institute of Biomedical Imaging and Bioengineering (R21 EB031317, principal investigator Markey). The funding agreement ensured the authors’ independence in designing the study, interpreting the data, and writing and publishing the report.

## ACKNOWLEDGMENTS

The authors would like to thank the patients who participated in this study and the team at the Behavior Research and Treatment Center for supporting this study. The authors also thank Dawn Chalaire of the MD Anderson Research Medical Library for her expertise in editing this article.

## ETHICAL APPROVAL

The institutional review board at The University of Texas MD Anderson Cancer Center reviewed and approved all aspects of this study (protocol number: PA17-0781). All images were selected by a board-certified reconstructive surgeon (G.P.R.) from our Breast Reconstruction Knowledgebase of more than 500 cancer survivors, which were collected prospectively as part of a previous study (National Institutes of Health [R01 CA143190]) approved by the institutional review board of The University of Texas MD Anderson Cancer Center protocol number 2010-0321.

## Supplementary Material


