# Supplementary material for: What Rankings, Ratings, and Utilities Do Breast Cancer Patients Place on Tissue- and Implant-based Breast Reconstruction?
Source: Plast Reconstr Surg Glob Open. 2025 May 1;13(5):e6749. doi: 10.1097/GOX.0000000000006749 (PMC12045546; doi:10.1097/GOX.0000000000006749)
Supplement: Supplementary file 1 [file gox-13-e6749-s001.pdf]

Supplementary Digital Content 1

Example image and health state cards (fronts and backs) for a woman who was overweight and had bilateral breast reconstruction.

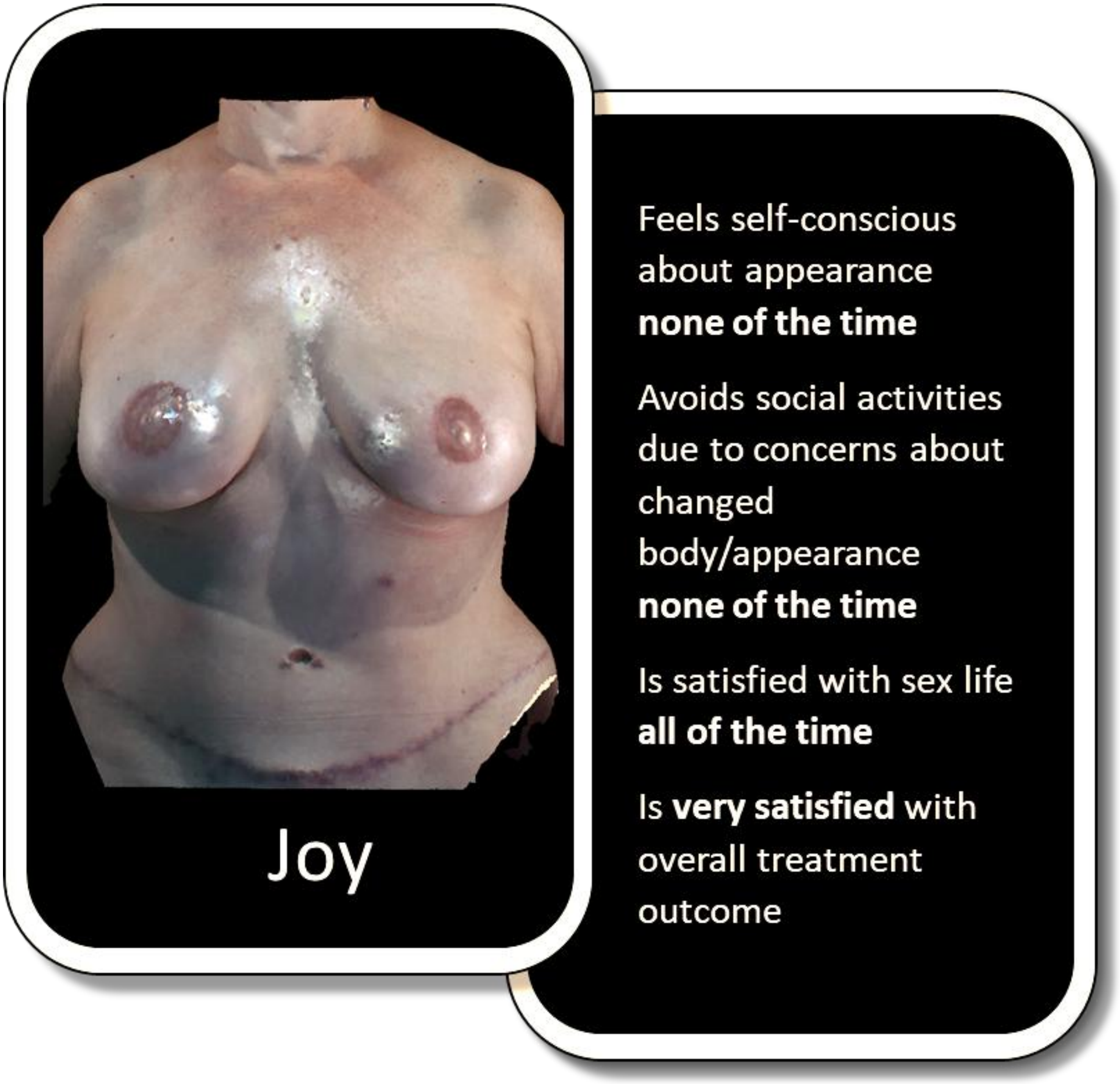

Excellent Tissue-based Reconstruction.

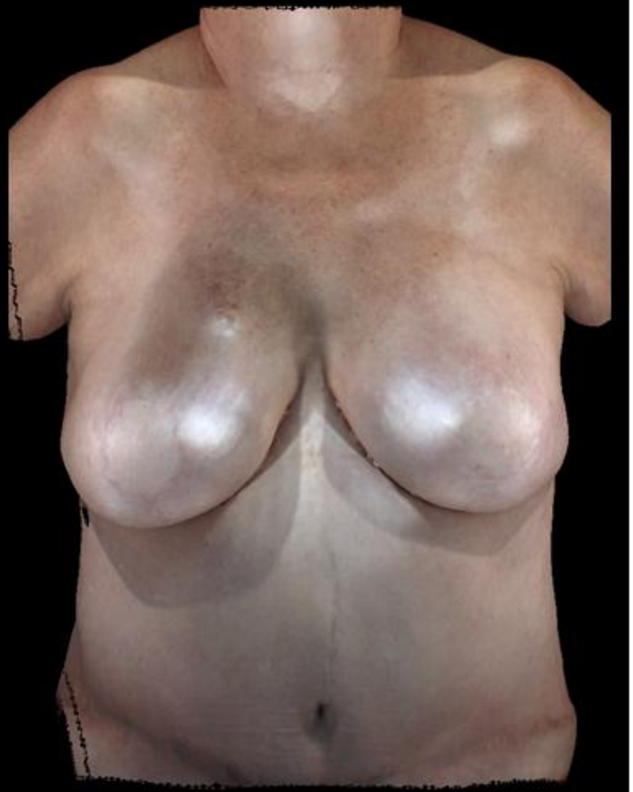

Sally

Feels self-conscious about appearance **a little of the time**

Avoids social activities due to concerns about changed body/appearance **a little of the time**

Is satisfied with sex life **most of the time**

Is **somewhat satisfied** with overall treatment outcome

Good Tissue-based Reconstruction.

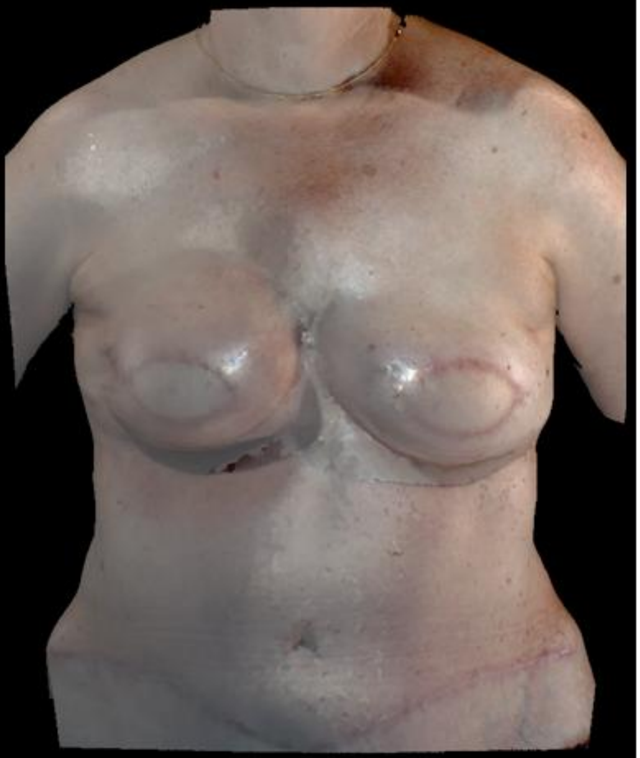

Kathy

Feels self-conscious about appearance **most of the time**

Avoids social activities due to concerns about changed body/appearance **most of the time**

Is satisfied with sex life **a little of the time**

Is **somewhat dissatisfied** with overall treatment outcome

Fair Tissue-based Reconstruction.

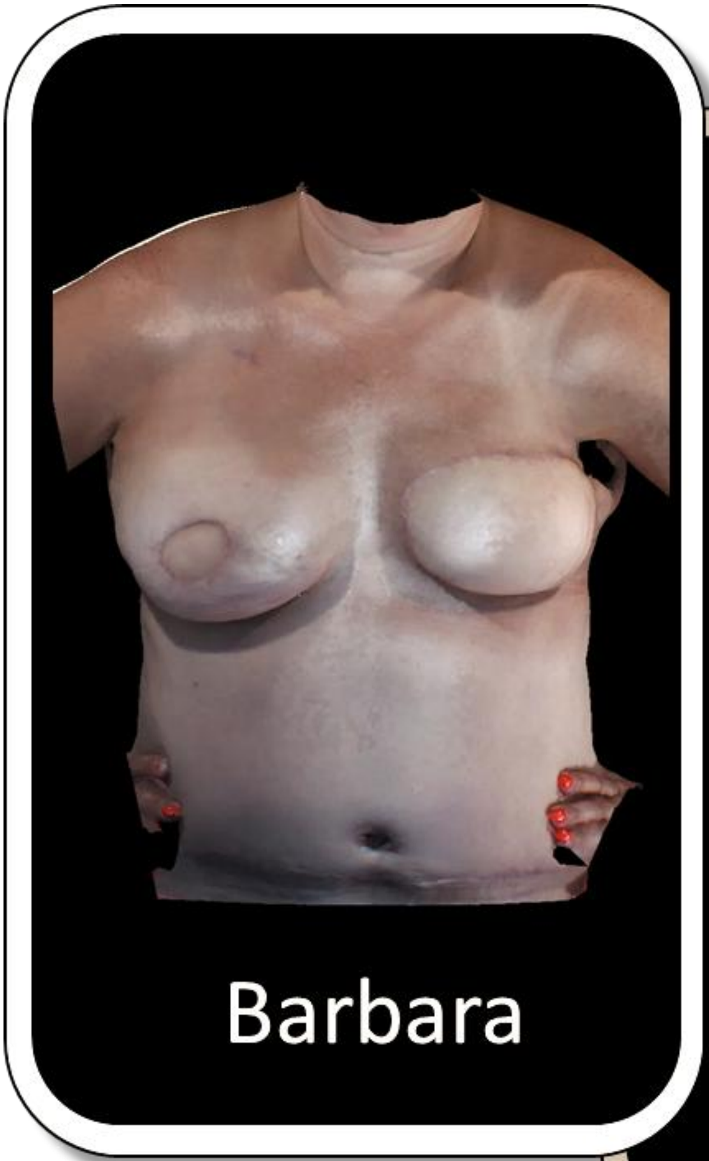

Feels self-conscious about appearance **all of the time**

Avoids social activities due to concerns about changed body/appearance **all of the time**

Is **not satisfied** with sex life

Is **very dissatisfied** with overall treatment outcome

Poor Tissue-based Reconstruction.

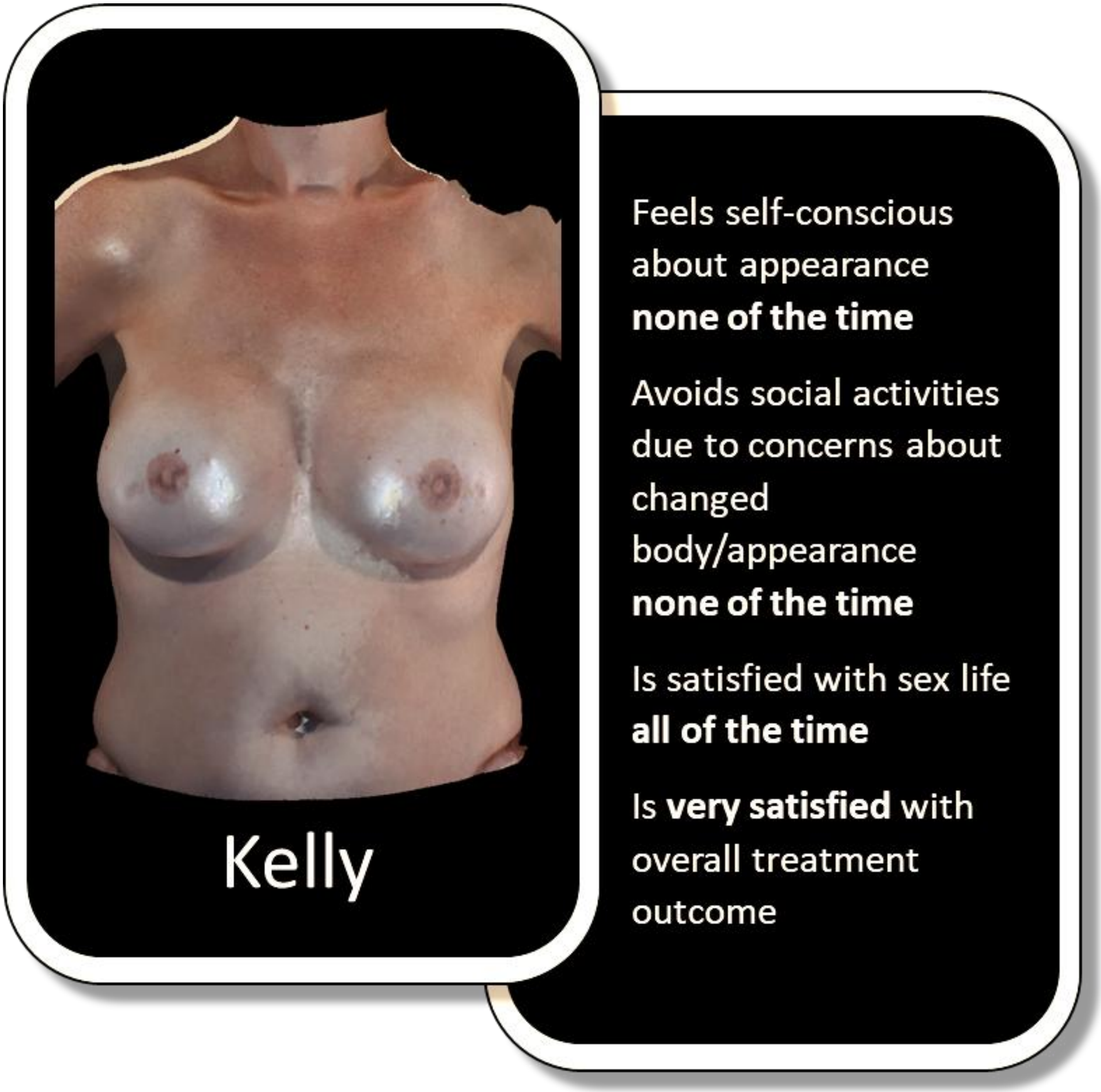

Excellent Implant-based Reconstruction.

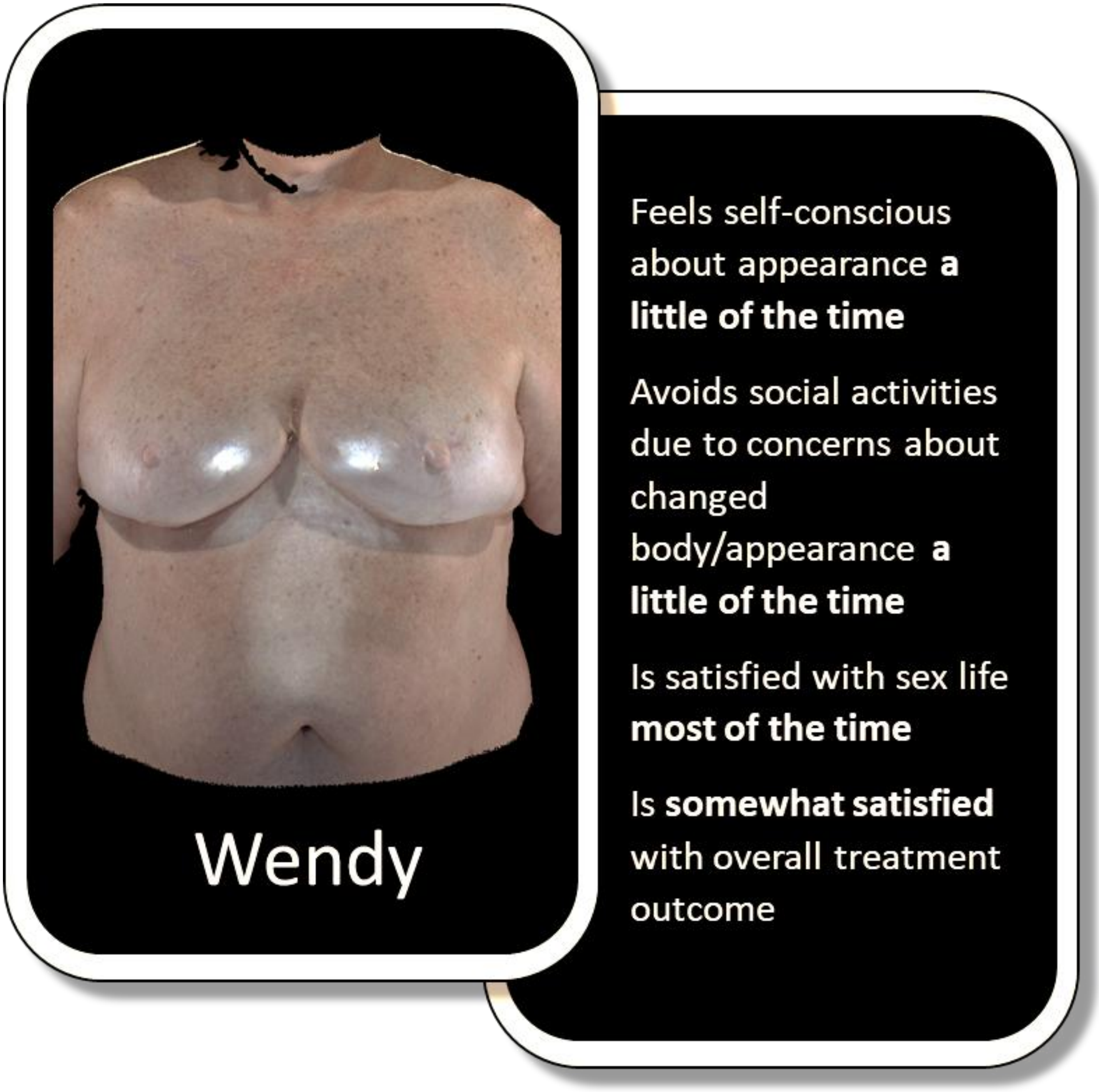

Good Implant-based Reconstruction.

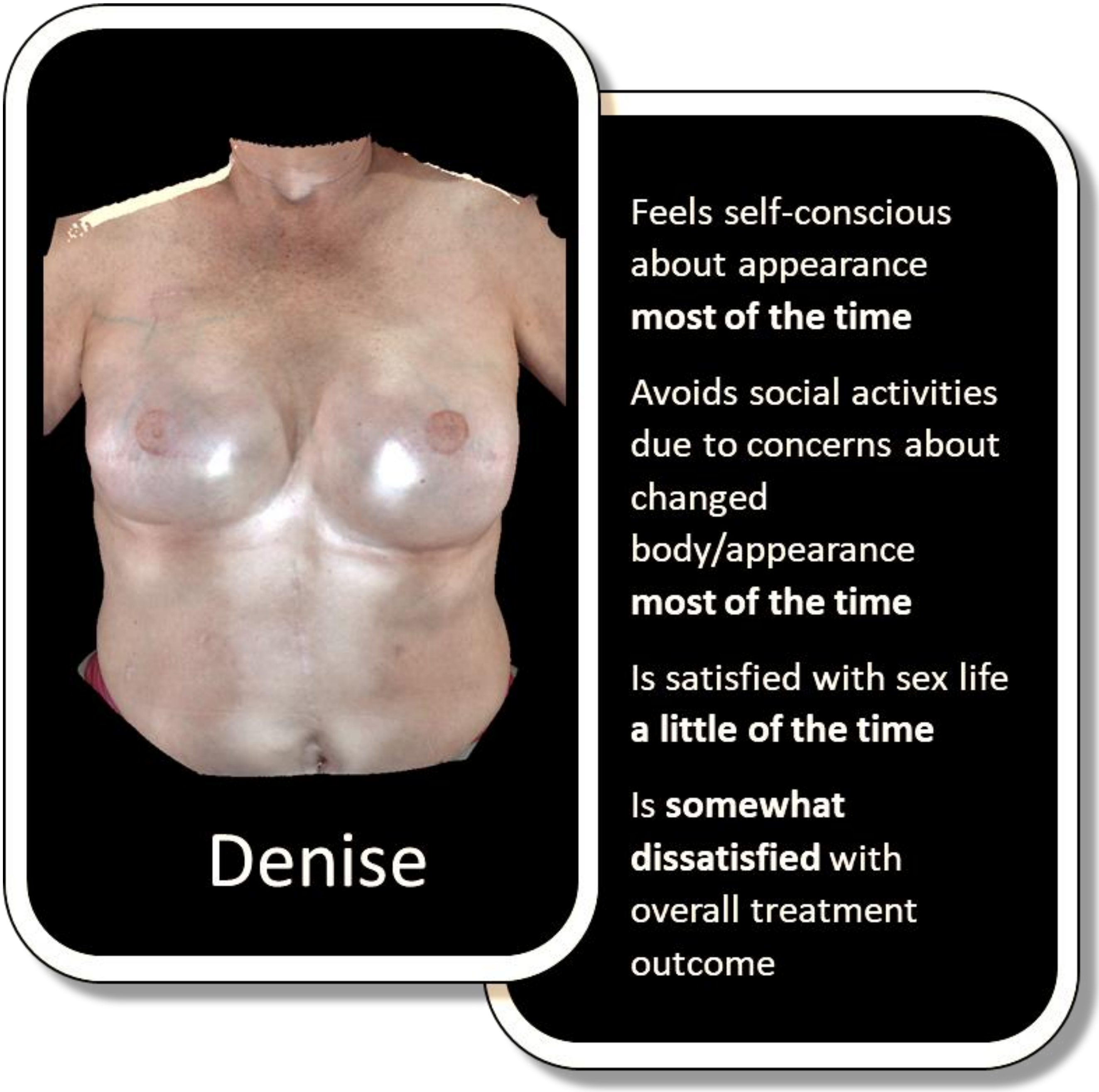

Fair Implant-based Reconstruction.

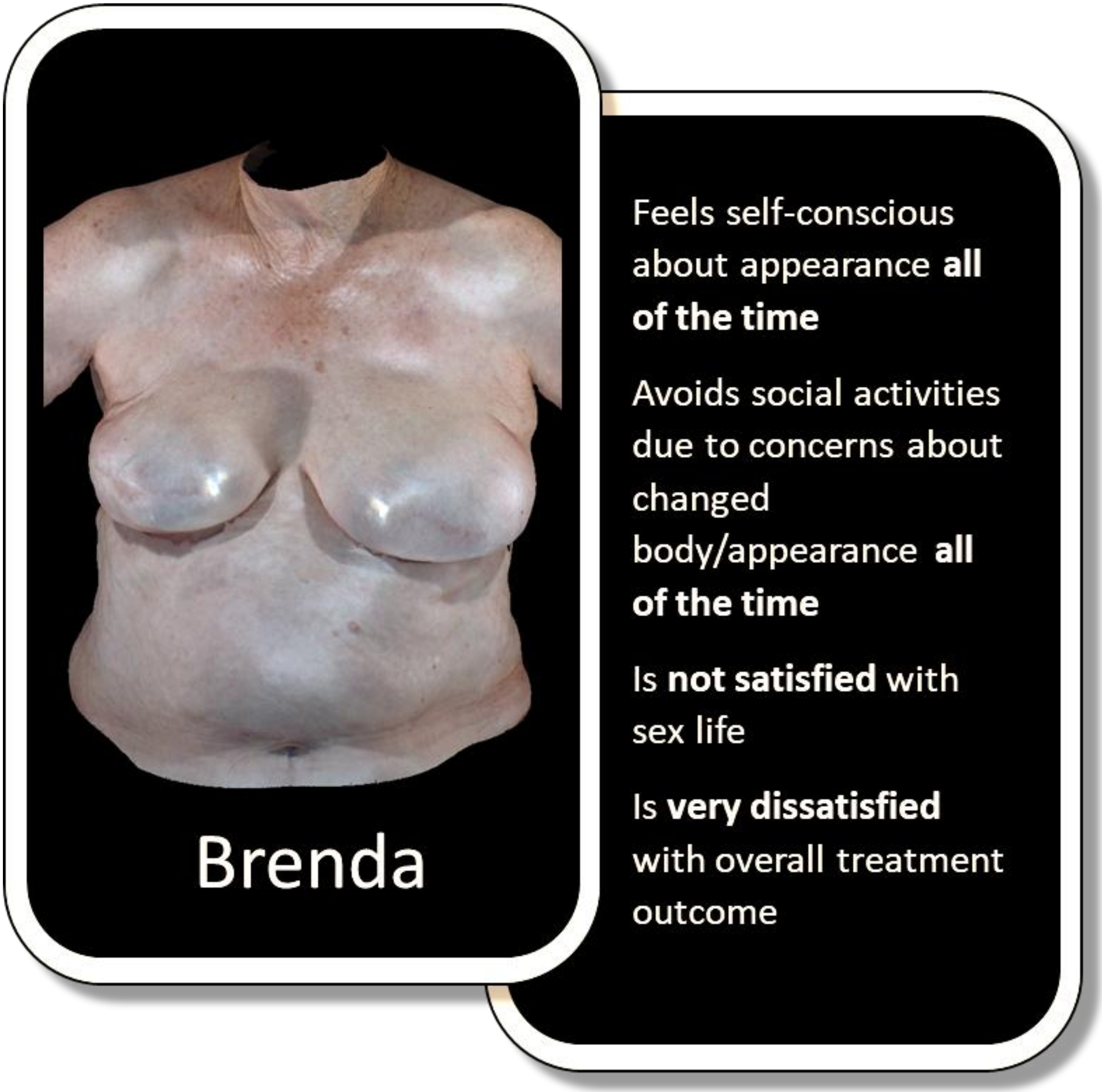

Poor Implant-based Reconstruction.

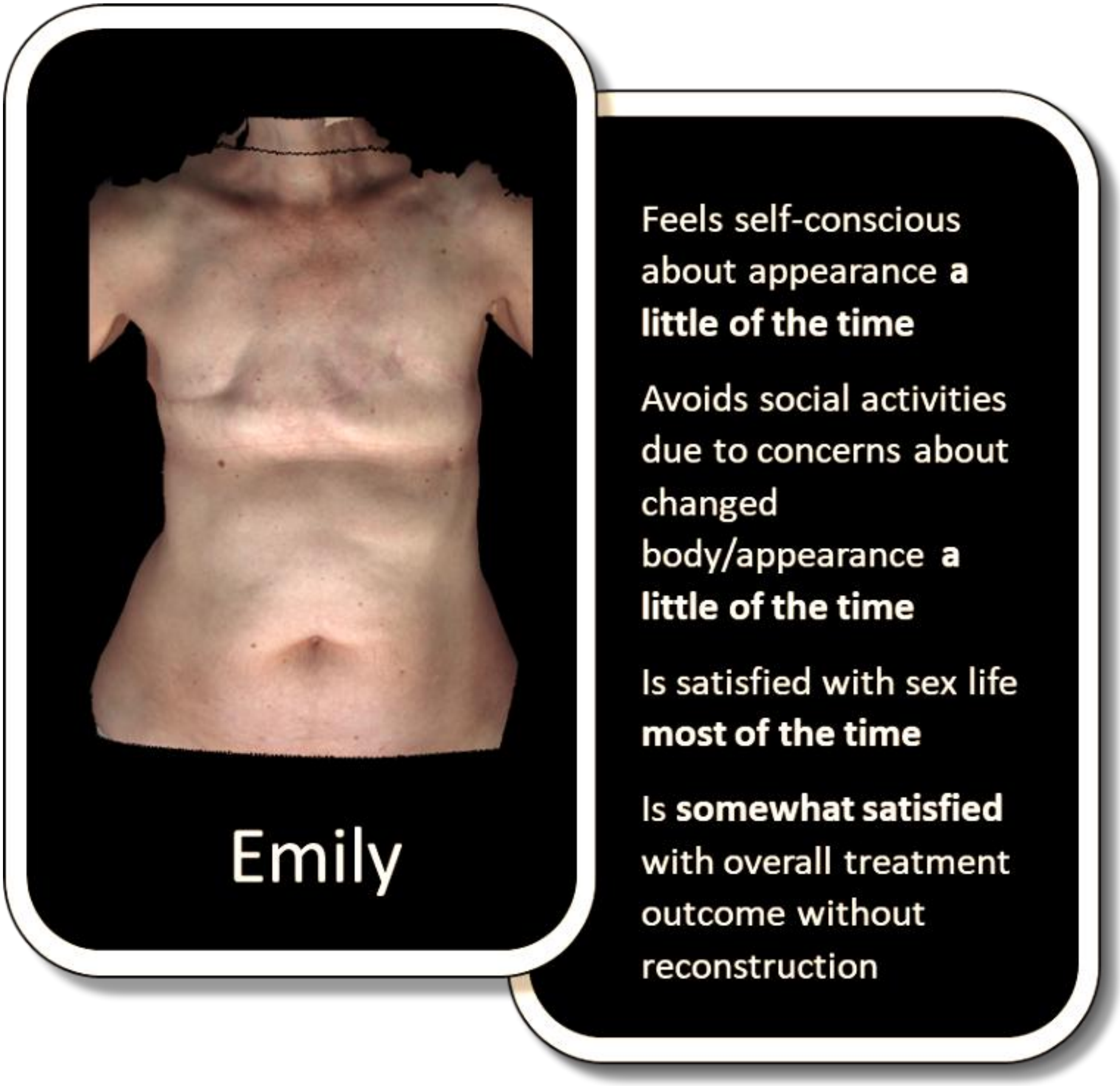

No reconstruction.
